# Public interest trends for Covid-19 and alignment with the disease trajectory: A time-series analysis of national-level data

**DOI:** 10.1371/journal.pdig.0000271

**Published:** 2023-06-09

**Authors:** Panayiotis D. Ziakas, Eleftherios Mylonakis

**Affiliations:** Department of Medicine, Houston Methodist Hospital, Houston, Texas, United States of America; Tsinghua University, CHINA

## Abstract

Data from web search engines have become a valuable adjunct in epidemiology and public health, specifically during epidemics. We aimed to explore the concordance of web search popularity for Covid-19 across 6 Western nations (United Kingdom, United States, France, Italy, Spain and Germany) and how timeline changes align with the pandemic waves, Covid-19 mortality, and incident case trajectories. We used the *Google Trends* tool for web-search popularity, and “Our World in Data” on Covid-19 reported cases, deaths, and administrative responses (measured by stringency index) to analyze country-level data. The *Google Trends* tool provides spatiotemporal data, scaled to a range of <1 (lowest relative popularity) to 100 (highest relative popularity), for the selected search terms, timeframe, and region. We used “coronavirus” and “covid” as search terms and set the timeframe up to November 12, 2022. We obtained multiple consecutive samples using the same terms to validate against sampling bias. We consolidated national-level incident cases and deaths weekly and transformed them to a range between 0 to 100 through the min-max normalization algorithm. We calculated the concordance of relative popularity rankings between regions, using the non-parametric Kendall’s W, which maps concordance between 0 (lack of agreement) to 1 (perfect match). We used a dynamic time-warping algorithm to explore the similarity between Covid-19 relative popularity, mortality, and incident case trajectories. This methodology can recognize the similarity of shapes between time-series through a distance optimization process. The peak popularity was recorded on March 2020, to be followed by a decline below 20% in the subsequent three months and a long-standing period of variation around that level. At the end of 2021, public interest spiked shortly to fade away to a low level of around 10%. This pattern was highly concordant across the six regions (Kendal’s W 0.88, p< .001). In dynamic time warping analysis, national-level public interest yielded a high similarity with the Covid-19 mortality trajectory (Similarity indices range 0.60–0.79). Instead, public interest was less similar with incident cases (0.50–0.76) and stringency index trajectories (0.33–0.64). We demonstrated that public interest is better intertwined with population mortality, rather than incident case trajectory and administrative responses. As the public interest in Covid-19 gradually subsides, these observations could help predict future public interest in pandemic events.

## Author summary

In this study, we used web search query data to study public interest for Covid-19 in the United States and major Western countries. We showed that after the initial shock of a new global pandemic, the relative public interest in Covid-19 declined, and the pattern of change was notably similar across the countries we studied. Furthermore, we explored similarities with the actual disease trajectory, regarding incidence, mortality and administrative responses, using a dynamic time-warping algorithm. The methodology allows for pattern recognition of time-series and can summarize similarity as a single measure of distance. We found that web search popularity for Covid-19 aligned better with the actual mortality trajectory and may mirror the pandemic’s course.

## Introduction

Public health information and research increasingly rely on internet sources of epidemiological data [1,2]. Search engine data have provided insights into the public’s perception of cancer epidemiology [3], infectious disease outbreaks [4], vaccination campaigns [5], and interest in prescribed medicines [6]. Google (Google Inc., Mountain View, CA, USA) is the most popular platform to explore public behavior and attracts the majority of web searches [7], and the *Google Trends* online tool can be used to study spatiotemporal data on search queries.

Three years have elapsed since the declaration of Covid-19 pandemic by the World Health Organization. By February 6, 2023, more than 671 million people were reported with Covid-19, and more than 6.8 million deaths were documented globally [8]. Aside from human losses, the socioeconomic impact of the pandemic is unprecedented [9]. In events of profound public interest, such as the Covid-19 pandemic, the study of web search behaviors could add to our understanding of the disease trajectory and serve as a prediction tool within the limits and reliability of digital epidemiology [10,11]. We used *Google Trends* as a proxy to monitor national public interest for Covid-19 in the United States and explored similarities in web search patterns with other Western countries. We aimed to study the association of public interest with the Covid-19 trajectory, including new cases, deaths, and administrative responses.

## Methods

A succinct methodological framework of the study is available in Table A in S1 Appendix.

### Data sources

#### Public interest in Covid-19

The *Google Trends* tool gives the time-varying output of the relative popularity of a specific search query, scaled to a range of <1 (lowest) to 100 (highest) for the selected time frame. We used “coronavirus” and “covid” as search terms and set the analysis timeframe beginning on January 1, 2020 and ending on November 12, 2022. We initially set the geographic region to "United States" and exported the pertinent data file to construct the time-series of relative popularity. We repeated the process for selected, highly populated Western countries, namely the United Kingdom (U.K.), France, Italy, Spain and Germany. Both authors have agreed on the list of countries to constrain bias related to multilingual search queries for countries using other than the Latin alphabet.

#### Covid-19 incidence and mortality

We used the "Our World in Data" database to extract country-level, time-series data on Covid-19 reported cases, deaths, and stringency index (up to October 30, 2022) [12]. These are open-source, open-access data under the Creative Commons BY license, allowing free use, distribution, and reproduction. We consolidated data weekly to minimize bias related to reporting policies, as the testing strategies and case notification differ between countries and local authorities. Moreover, the lag between testing to a positive result notification and the day of testing from the symptom onset may also vary within a country. Adding retrospective data consolidation and reporting delays, the number of new events daily does not capture the contagion trajectory, while weekly pooling will reflect more accurately the contagion process [12]. Weekly data on cases and deaths were transformed (scaled) to a range between 0 to 100 using the min-max normalization algorithm, which preserves the distribution of the original data, does not affect the importance of outliers [13], and, importantly, will allow the direct comparison with web search popularity data.

#### Administrative response to Covid-19

To describe the similarity between web search popularity and a country’s administrative response, we used the Covid-19 Government Response Stringency Index by the University of Oxford [14]. This index combines 9 specific indicators of response of an ordinal scale into a single additive composite measure, normalized to vary from 0 to 100. The nine components include school and workplace closings, restrictions on gatherings and public events, public transport restrictions, movement restrictions and stay-at-home policies, international travel controls, and public information campaigns [14].

The compiled timeseries data on new cases, deaths, and administrative responses were used to track the pandemic trajectory, herein defined as “Covid-19 trajectory”.

### Main outcome measures

#### Concordance of public interest between countries

For each country, we used a *Google Trends* dataset of web search popularity at the national level. We measured the concordance of rankings across the six selected countries using the non-parametric Kendall’s W. The test statistic spans 0 to 1, where 1 suggests a perfect match, and 0 indicates a lack of agreement [15].

#### Validation of web search output against sampling bias

*Google Trends* data are anonymized. A random sample to match geography and time is drawn from the anonymous database following the introduction of a search term in order to generate the relative popularity report. The results are cached for 24h to be deleted afterward, so a new search with the exact query will recall a new random sample. Therefore, sampling bias may differentiate the output of the subsequent searches. We obtained 10 consecutive data outputs after cache renewal between February 3, 2023 to February 12, 2023 using the same criteria and timeframe to validate our findings. We used the first output for the analysis and the remaining outputs for validation (relative popularity available in S1 Data).

#### Comparison between public interest and Covid-19 trajectory

The occurrence of new cases and related deaths are measures of outstanding importance for epidemiology and public health to describe the pandemic trajectory. Cases and related mortality vary by time and region to form a longitudinal profile unique for each region and can serve as a reference to study similarities with other measures of interest by visual assessment and timeseries analysis [16]. We compared the relative web search popularity with the Covid-19 trajectory, namely weekly mortality, incident cases, and stringency index. The similarity was mapped at the range of 0 to 1, with 0 denoting lack of any similarity (perfect dissimilarity) and 1 denoting perfect similarity. We used a whole matching process (along the entire series length) to compute similarity, using a weighted version of L₁ (Manhattan) distance, the Canberra distance. The latter distance metric was preferred because it is mapped within a 0 to 1 boundary and performs better with non-negative numbers and ranked lists [17,18]. We selected a dynamic time-warping algorithm for the optimal alignment of time-series, which allows us to recognize the similarity of shapes and is robust to outliers, shifting, or scaling. This methodology permits an elastic adjustment of time to identify similarities and time-shifting patterns between time-series [19]. The similarity index was calculated by subtracting the mean distance between time series from unity. We limited warping time up to 8 weeks to avoid unconstrained alignment beyond epidemiologically relevant time lags. We based the decision on Covid-19 deaths and simulation data (available in Figure A in S1 Appendix) since mortality, the most lagged measure of the pandemic trajectory, follows three or more weeks after case confirmation with extra delays in documentation and reporting [20,21]. The “dtw Package” [22] was used in R for data analysis (R code with data available in S2 Data) [23].

## Results

### High concordance of public interest between countries

**Fig 1:**
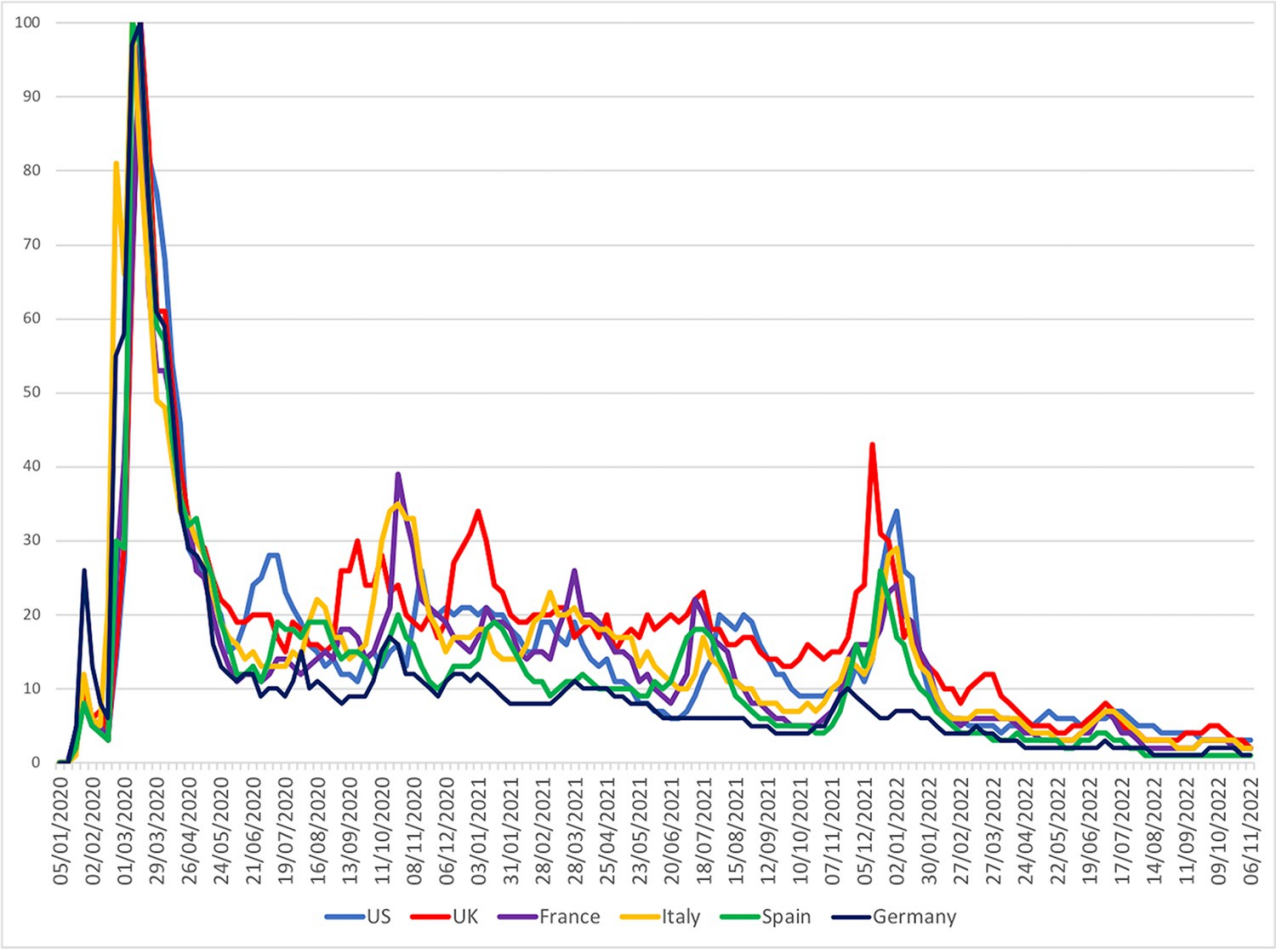
Web-search relative popularity trends as a proxy of public interest for Covid-19. National-level timeseries (United States, United Kingdom, France, Italy, Spain and Germany), weekly intervals. High concordance of rankings (Kendal’s W 0.88, p<0.001).

On the visual assessment of the relative popularity of Covid-19 across national-level data in Fig 1, striking similarities appear. More specifically, the peak popularity was recorded in March 2020. In the subsequent three months, popularity declined sharply below 20%, followed by a long-standing period of variation around that level. The latter lasted until the end of 2021, interrupted by a sharp spike in relative popularity. After that, web search popularity declined to maintain a low activity, around 10% until the end of the observation period, with Germany having slightly lower levels long-term.

There was a high level of concordance between the six selected countries regarding Covid-19 public interest on weekly assessments (Kendal’s W 0.88, p< .001), shown in **Fig 1**. In the less conservative setting of monthly data comparison, the test also yielded 0.88 (p< .001).

### Validation with multiple sampling

After multiple sampling, the concordance of public interest within and between regions was stable, indicating minimal sampling bias. There was 99.7%-99.8% concordance within regions for the timeline popularity patterns and 87.3%-87.7% between regions of interest (Table B in S1 Appendix).

### Public interest aligns with Covid-19 trajectory

**Fig 2:**
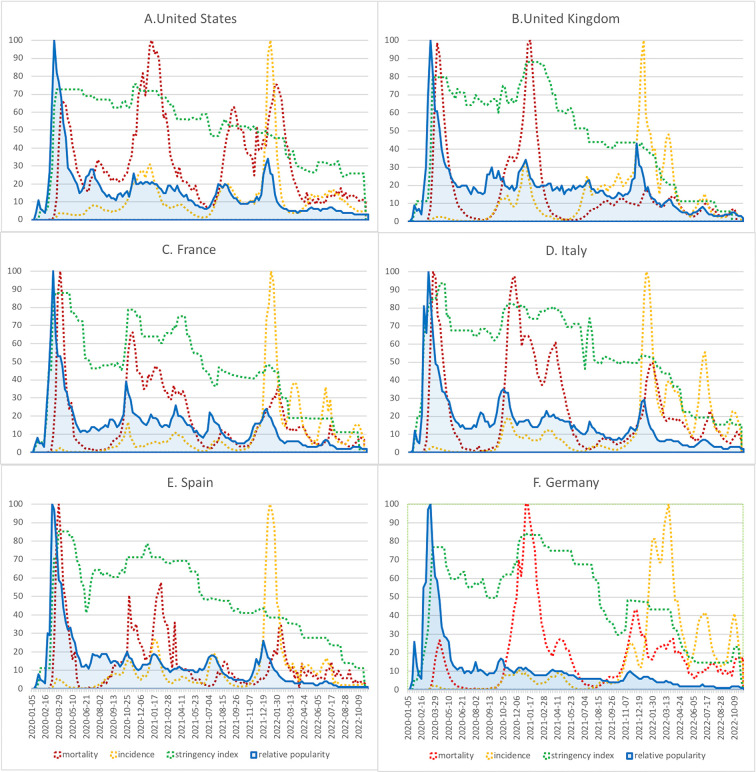
An overlay plot for the relative web-search popularity, Covid-19 incident cases, mortality, and stringency index trajectories, stratified by country of origin. Incidence and mortality data after scaling with min-max normalization algorithm (**A**). United States (**B**). United Kingdom (**C**). France (**D**). Italy (**E**). Spain (**F**). Germany.

On visual assessment (**Fig 2**), the web-search popularity patterns intuitively seem a better match to the Covid-19 mortality trajectories rather than the new cases and stringency index trajectories. Specifically, the peak popularity noted in March 2020 after the pandemic broke out, preceded the mortality peak of the initial wave by 2–4 weeks. Relative popularity oscillated around 20% after that, being disproportionate to the mortality trajectory for the subsequent Covid-19 waves. However, spikes in relative popularity were noted up to 2 weeks before the peak in incidence and 1–8 weeks earlier before mortality peaks during late 2020 and early 2021 Covid-19 waves. By the end of 2021 and early 2022, when omicron variants appeared, the public interest sparked temporarily 1–3 weeks before the wave peak and 3–8 weeks before the mortality peak. In **Table 1** we show the dynamic time warping estimates. The analysis yielded similarity indices ranging from 0.60 to 0.79 between web-search popularity and mortality trajectories (time series alignment available in Figure B in S1 Appendix). With the exception of Germany, all estimates were above 0.70. For web-search popularity and incidence cases trajectories, similarity ranged between 0.50 to 0.76 (time series alignment available in Figure C in S1 Appendix). Finally, web-search popularity and stringency index patterns had similarity estimates between 0.33 to 0.64 (time series alignment available in Figure D in S1 Appendix). Public interest had the weakest match with the stringency index trajectory.

**Table 1:** Similarity indices between relative web search popularity and Covid-19 time-series.

Similarity with the relative popularity	Mortality trajectory	Incident cases trajectory	Stringency Index (S.I.) trajectory	Best match
United States	0.74	0.76	0.47	incidence by narrow margin
United Kingdom	0.79	0.68	0.64	mortality
France	0.78	0.58	0.53	mortality
Italy	0.72	0.53	0.44	mortality
Spain	0.75	0.69	0.38	mortality
Germany	0.60	0.50	0.33	mortality

Annotation: indices estimated with dynamic time warping algorithm; 0 = no similarity, 1 = perfect similarity; constrained warping for w = 8 weeks as the maximal amount the warping path is allowed to deviate

## Discussion

The public responded with a voluminous interest in the emergence of a new public health threat, Covid-19 after the World Health Organization formally declared the pandemic. In the early months of the pandemic, web search popularity was high but gradually subsided to oscillate around moderate levels. The emergence and dominance of omicron variants, beginning at the end of 2021 and early 2022, temporarily revived web search popularity for Covid-19. After that, relative popularity reset to low levels, which persisted throughout 2022. The timeline of changes in web search popularity has been strikingly similar across the countries we studied.

We found that web search popularity patterns resemble the Covid-19 contagion growth, and spikes in public interest may appear weeks before wave peaks. However, it appears that after the initial shock, the web-search popularity has lost potency, with the exception of omicron outbreak. This is perhaps expected as people become gradually desensitized to the onset of the pandemic and naturally turn their interest to the conditions in healthcare institutions, emergency service pressure and the perceived personal risk of death. Nevertheless, relative popularity aligned preferentially with the death toll of the pandemic rather than the number of cases or a government’s restrictions and public information policies. This is expected since mortality is a more robust epidemiologic measure to describe the pandemic burden compared to confirmed incident cases, which are grossly moderated by testing and reporting biases [21]. Furthermore, the recent decline in Covid-19 testing has led to underestimating the actual case trajectory, probably more than ever. Besides objective, psychological factors may have also influenced web search popularity for Covid-19 and attenuated public interest. Significant knowledge has been accumulated for the past years of the pandemic, and populations have become increasingly acquainted with the virus. The aversion of uncertainty has led to declining interest, specifically during the last year when it became clear that the virus will not be vanquished but stay with us for long, with mortality restricted by previous exposure, vaccination, and specific treatment [24–27]. For Germany, the combination of exemplary handling of the early pandemic phases [28] and the long-standing, more favorable public view of the country’s response [29], may have shaped relative interest in Covid-19 somewhat lower than its peers and attenuated popularity-mortality similarity.

After vaccination and/or infection, a large part of the public returned quickly to their pre-Covid-19 era routine. The virus evolves to a better fit while populations adapt more efficiently to withstand illness and downgrade their perception of the "threat" imposed by the disease [30]. However, people fatigued by the pandemic may have still responded disproportionally to the omicron surge compared to previous waves [31]. Finally, the lifting of remaining restrictions worldwide in early 2022 has officially signaled the return to a state of normality, which again limited relative interest in the pandemic. Government and public interest have narrowed to preventing severe outcomes, as most arguments in favor of population restrictions are gone [32]. Through historical times, population attitudes toward pandemics may not have changed dramatically. Societies initially respond with great interest to a new pandemic, sometimes with panic, superstition, or conspiracy theories. Afterward, as the pandemic fades away, populations become less interested and concerned [33,34].

Case rates and mortality are critical epidemiologic measures to describe pandemic growth but they lag behind the actual status of the pandemic, with mortality being the most lagging metric, and their availability relies upon administrative authorities and healthcare organizations. Web search behavior could provide a dynamic, real-time proxy of public interest, that is readily accessible even before incidence and mortality data become available. In the context of the Covid-19 pandemic, volatility in web-search popularity was an early sign of a new wave, and we found that it was followed by a rise in incident cases and mortality. Interestingly, public interest in Covid-19 was not related to central or governmental response to the pandemic, suggesting that it could be a useful metric irrespective of restrictive measures.

Moreover, our study suggests that changes in relative popularity could serve as early indicators of new pandemic waves, but they are unlikely to capture the incidence and mortality highs of the upcoming wave. Our working hypothesis is that there are at least two contributing factors that prohibit higher agreement between public interest and pandemic metrics. First, population interest is higher during the early stages of the pandemic, and interest inevitably diminishes following expanded knowledge, prolonged exposure, and a downgraded perception of risk. Second, bias related to underreporting of cases or deaths could affect this correlation and vary by time and region. Despite these inevitable constraints, the availability, reproducibility, and satisfactory approximation of the Covid-19 growth trajectory render web-search popularity of value to monitoring current and future pandemics aside from standard epidemiologic measures.

### Limitations

The use of web search popularity is a valuable tool to monitor public interest within limits imposed by research protocols, the *Google Trends* methodology itself, and the public understanding of the target condition. In brief, research with *Google Trends* lacks a standardized protocol, and findings across studies may vary regarding reproducibility [35]. Furthermore, the methodology of the online tool is amended periodically by the provider, and the undisclosed changes may affect findings. *Google Trends* may mirror public interest for a general research topic, but qualitative analysis requires in-depth data mining, organization, and cleaning using multiple terms, as previously noted [36,37]. Poor familiarity with the target condition may underestimate the actual disease burden, as in 2009 and 2010, when *Google Trends* missed the first pandemic wave of influenza in the U.S. but accurately mirrored the second wave [38]. Disproportionate media coverage may magnify web search popularity for rare target conditions, e.g., the Ebola epidemic in Africa boosted public interest in the United States in the absence of an epidemic in the U.S. [39]. Covid-19 cases and deaths are submitted by national governments, aiming to report confirmed events to secure a standardized comparison. Figures are subject to periodic correction, and slightly different criteria may exist across countries for definition and registration [12]. Case documentation varies by region, disease severity and time, with the actual incidence generally underestimated. Heterogeneity in testing and case documentation may have increased mismatch with the public interest, particularly during the early pandemic waves, when both public interest and mortality were high, but access to testing resources was limited. Documentation of death data is less biased but is the most lagged measure with delays in reporting [21,40,41]. For example, the U.S. CDC estimated that only a quarter of Covid-19 infections and 1 out of 1.32 related deaths were registered [42]. As a final remark, we analyzed data in a subset of developed Western countries where population internet use reached 90% in 2021 [43]. Public interest is an inclusive term to describe the attention or concern that the general public has on a specific topic or event. This public interest is reflected in public discussions, media coverage, and web searches. Among these indicators of public interest, web searches are considered a reliable proxy for public interest and universal internet access enhances data reproducibility and the robustness of findings. For less developed digital environments and multiple language queries, the present findings and associations may not apply.

Monitoring internet search popularity can go beyond tracking public interest to forewarn for future disease outbreaks and pandemics. Specifically, it could contribute to early detection and surveillance, and increase preparedness by authorities and public health officials to track and contain disease spread, as well as provide data for the allocation of appropriate resources. Geographic monitoring could identify the most affected areas and guide public health interventions, including vaccines and treatment distribution, personnel and hospital bed allocation, community measures, and public messaging. Moreover, the timeline of web search popularity could provide insights into seasonal fluctuation of an epidemic disease, the emergence of a new strain, or the changes in public interest following the introduction of community measures and vaccination campaigns.

## Conclusions

At a national level, the web search interest for Covid-19 peaked with the emergence of the pandemic, with relative popularity declining in the long run, and short term-changes affected by the emergence of new variants, in particular omicron variants. This pattern was notably similar across a sample of Western countries. Driven by objective factors and public behavior toward the pandemic, web search popularity has mirrored the course of the pandemic and was a more accurate impression of the mortality trajectory rather than infection rate.

## Supporting information

S1 Appendix **Table A.** Methodological framework of the study**; Table B.** Concordance of relative public interest for Covid-19; **Figure A.** Popularity-mortality similarity estimates over warping time, simulation data**; Figure B.** Covid-19 relative popularity *vs*. mortality**; Figure C.** Covid-19 relative popularity *vs*. incidence; **Figure D.** Covid-19 relative popularity *vs*. stringency index.
(DOCX)

S1 Data Relative popularity data. (XLSX)

S2 Data Example of R code with data for calculations. (R)
