# Supplementary material for: Public interest trends for Covid-19 and alignment with the disease trajectory: A time-series analysis of national-level data
Source: PLOS Digit Health. 2023 Jun 9;2(6):e0000271. doi: 10.1371/journal.pdig.0000271 (PMC10255932; doi:10.1371/journal.pdig.0000271)

**S1 Appendix**

The authors have provided this appendix to give readers additional information about their work. Supplement to: “Public interest trends for Covid-19 and alignment with the disease trajectory: A time-series analysis of national-level data”, authored by P.D. Ziakas & E. Mylonakis.

**Table A: Methodological framework of the study**

| Purpose | To monitor changes in the popularity of Covid-19 in the United States and Western countries; to validate popularity patterns and trends; to associate popularity with the actual disease trajectory |
| --- | --- |
| Period Searched | 3-year span; Beginning January 1^st^ , 2020; end November 12^h^ , 2022 |
| Time division | Weekly intervals; monthly intervals for sensitivity analysis |
| Geographic division | United States, United Kingdom; France, Italy, Spain; Germany nationwide coverage |
| Web data source | Google Trends portal |
| Query category | All categories |
| Query combination used | combinations used: covid+coronavirus  A "+" sign between terms corresponds to an "OR" Boolean operator in Google Trends |
| Search rationale | To compare the relative web-search popularity for Covid-19 across countries of interest; to find similarities with the contagion trajectory, namely mortality and incident cases |
| Primary findings | Relative web-search popularity for Covid-19; concordance across countries; similarity with the contagion process |
| Analysis type | Time series analysis; dynamic time warping to assess the similarity of relative public interest with Covid-19 trajectory, mapped between 0 (lack of any similarity) to 1 (perfect similarity) |
| Comparison data | - Mortality from Covid-19, weekly data; trajectory scaled from 0-100 (min-max normalization algorithm) |
|  | - Incident cases with Covid-19, weekly data; trajectory scaled from 0-100 (min-max normalization algorithm) |
|  | -Government response stringency index ranging from 0-100 (100 is the strictest response); compiles nine indices on measures and public information for Covid-19 |

**Table B: Concordance of relative public interest for Covid-19**. Validation after multiple sampling (n=10)

| **Kendal’s W**^a^ |  |  |
| --- | --- | --- |
|  | **Same region estimates** | |
| United States | 0.998 | p< .001 |
| United Kingdom | 0.997 | p< .001 |
| France | 0.998 | p< .001 |
| Italy | 0.998 | p< .001 |
| Spain | 0.998 | p <.001 |
| Germany | 0.997 | p <.001 |
|  | **Between regions estimate** | |
| All six regions (median-range)^b^ | 0.875 | 0.873-0.877 |

^a^  0 (for lack of any concordance) to 1 (for prefect concordance)

^b^ all p-values <0.001

**Figure A. Popularity-mortality similarity estimates over warping time, simulation data**. Warping time was set to 8 weeks (w=8) for the main analysis to prevent unconstrained alignment beyond epidemiologically reasonable time.

**Figure B. Covid-19 relative popularity ( black solid line) *vs*, mortality ( red dashed line).** Time-series alignment with dynamic time-warping algorithm.
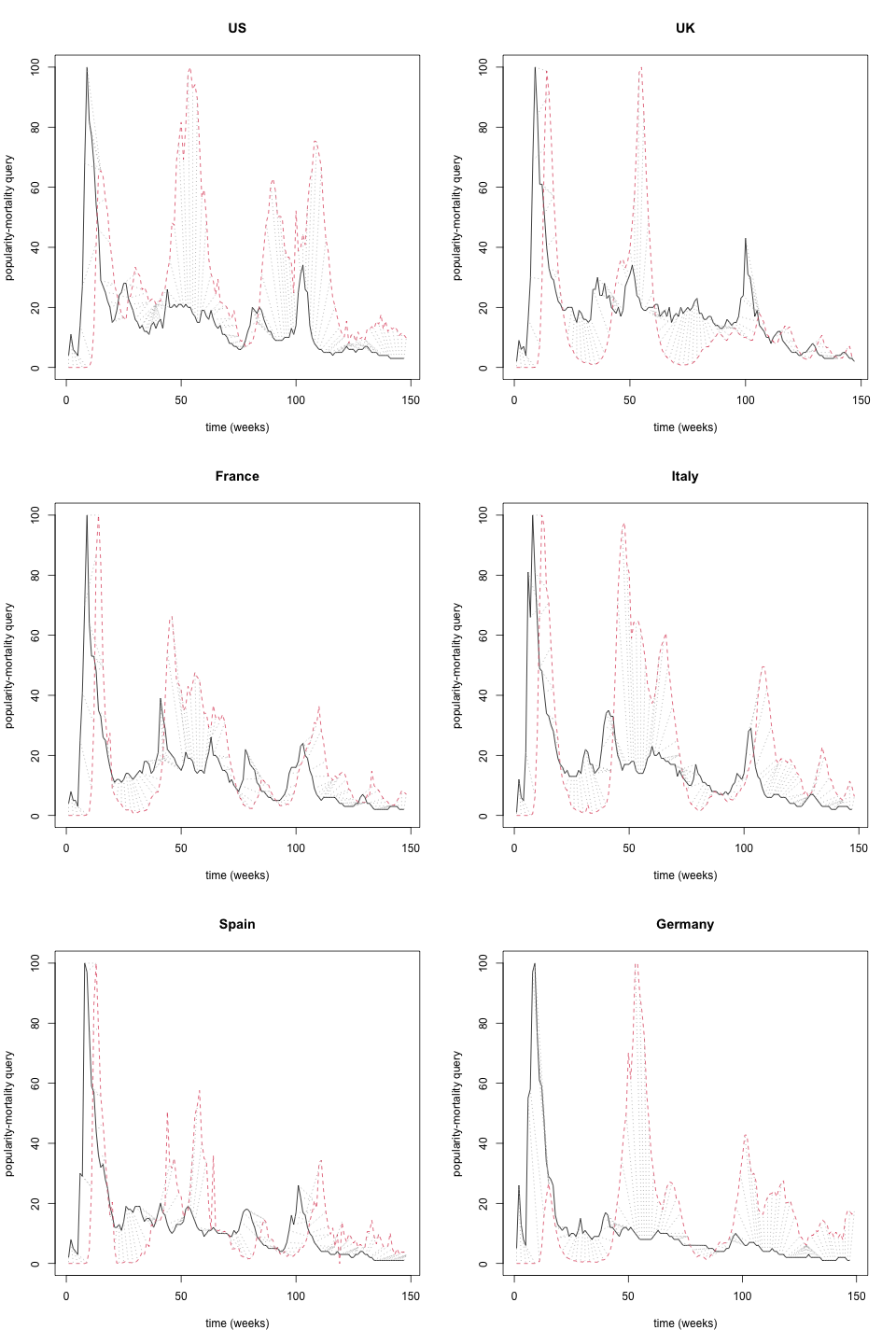


**Figure C. Covid-19 relative popularity ( black solid line) *vs*, incidence ( red dashed line).** Time-series alignment with dynamic time-warping algorithm.


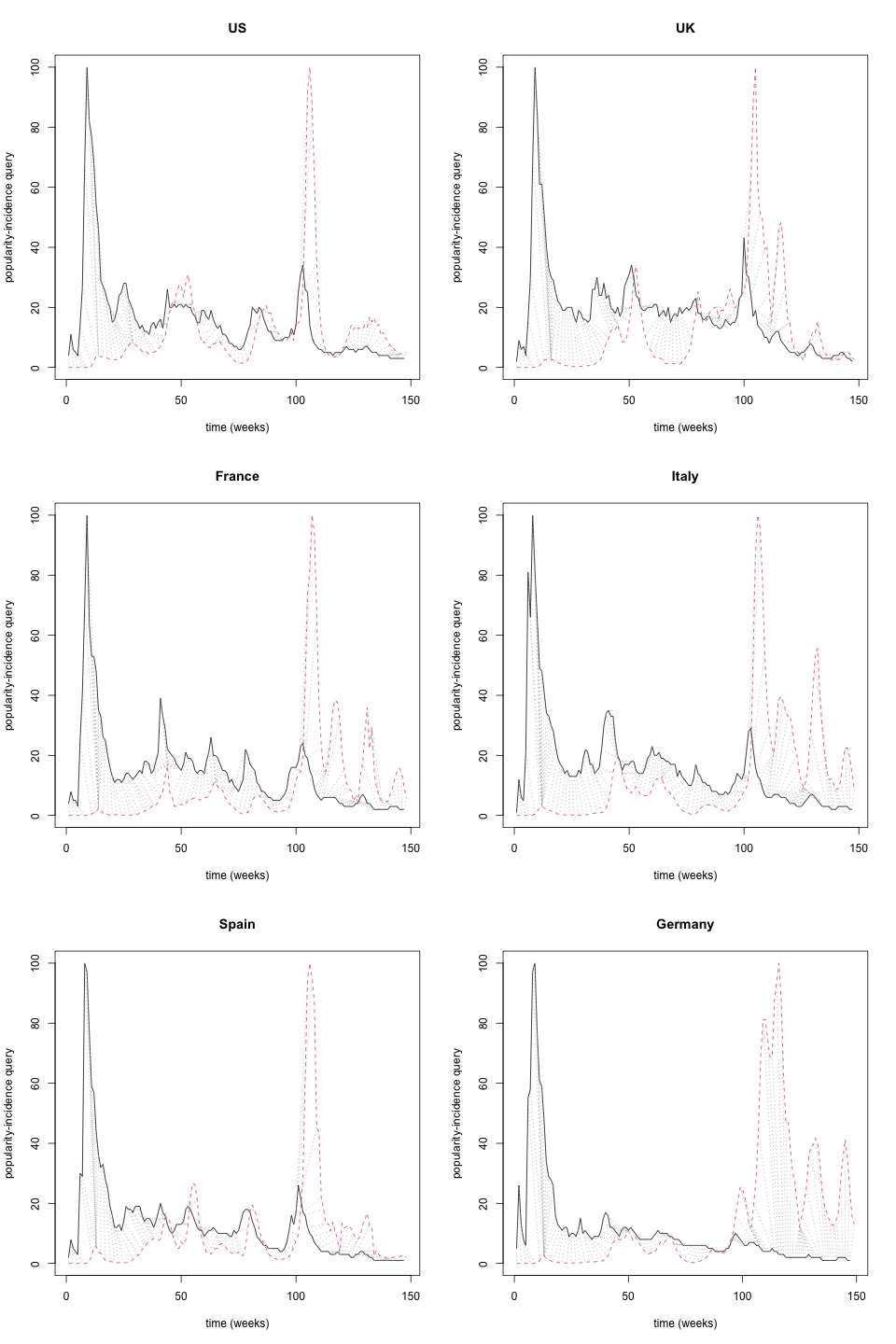


**Figure D. Covid-19 relative popularity ( black solid line) *vs*, stringency index ( red dashed line).** Time-series alignment with dynamic time-warping algorithm.


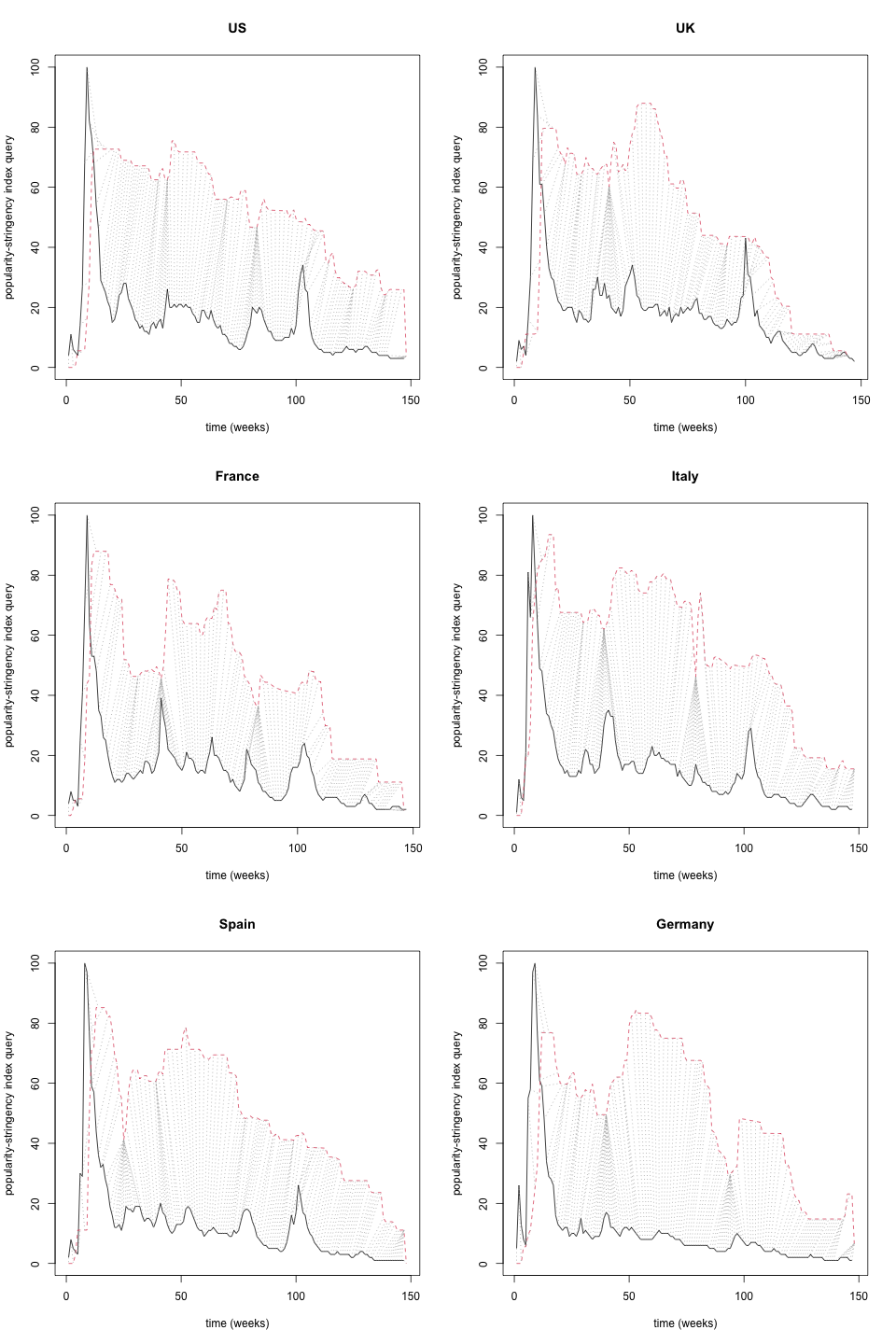

Supplement: S1 Appendix — Table A. Methodological framework of the study; Table B. Concordance of relative public interest for Covid-19; Figure A. Popularity-mortality similarity estimates over warping time, simulation data; Figure B. Covid-19 relative popularity vs. mortality; Figure C. Covid-19 relative popularity vs. incidence; Figure D. Covid-19 relative popularity vs. stringency index. (DOCX) [file pdig.0000271.s001.docx]
